# Salivary proteomics in patients with fixed orthodontic appliances and Invisalign treatment

**DOI:** 10.1016/j.identj.2025.103971

**Published:** 2025-11-08

**Authors:** Rosarin Chanpichai, Sittiruk Roytrakul, Sittichai Koontongkaew

**Affiliations:** a International College of Dentistry, Walailak University, Bangkok, Thailand; b National Center for Genetic Engineering and Biotechnology, National Science and Technology Development Agency, Pathum Thani, Thailand; c Faculty of Dentistry, Western University (Wacharaphol Campus) 4 Moo11, Pathum Thani, Thailand

**Keywords:** Orthodontic tooth movement, Salivary protein biomarkers, Fixed orthodontic appliances, Invisalign, Proteomic analysis, CD19, FAM20C, GNRHR, Liquid chromatography-tandem mass spectrometry

## Abstract

**Objectives:**

Early detection of biological changes is crucial for effective monitoring of orthodontic tooth movement (OTM). Research that elucidates the connection between OTM and salivary proteomic profiles remains limited. This cross-sectional study aimed to compare salivary protein profiles between fixed orthodontic appliances (FOA) and Invisalign (IN) treatments.

**Methods:**

Unstimulated saliva was collected from 17 patients treated with FOA and 6 patients treated with IN before treatment (T0), and at 3 (T1) and 6 (T2) months after therapy initiation. Salivary proteomes were analysed using liquid chromatography-tandem mass spectrometry, followed by label-free protein quantification and identification with MaxQuant. Heatmap visualisation, Partial Least Squares Discriminant Analysis (PLS-DA), and ANOVA were performed using MetaboAnalyst (*P* ≤ .05) to identify differentially expressed proteins (DEPs). Gene Ontology enrichment analysis and Protein-Protein Interaction (PPI) network analysis were conducted on the identified DEPs. *Results:* PLS-DA demonstrated clear separation between T0, T1, and T2 in both groups. From 17,603 DEPs, 12 and 50 top proteins were identified between FOA and IN at T1 and T2, respectively. Three DEPs—CD19, FAM20C, and GNRHR—were identified in both groups. Their expression levels significantly decreased (*P* < .05) at T1 and T2 within each group, with no significant differences in CD19 and FAM20C between the 2 groups at T0, T1, and T2. PPI revealed associations between these proteins and OTM-related proteins, including fibronectin, RANK, osteopontin, dentine sialophosphoprotein, SOX9, alkaline phosphatase, collagen alpha-1, and collagen alpha-2.

**Conclusion:**

CD19, FAM20C, and GNRHR are involved in OTM. *Clinical relevance:* Monitoring of CD19, FAM20C, and GNRHR expression during OTM may provide valuable biomarkers for predicting progression and outcomes.

## Graphical abstract

**Figure:**
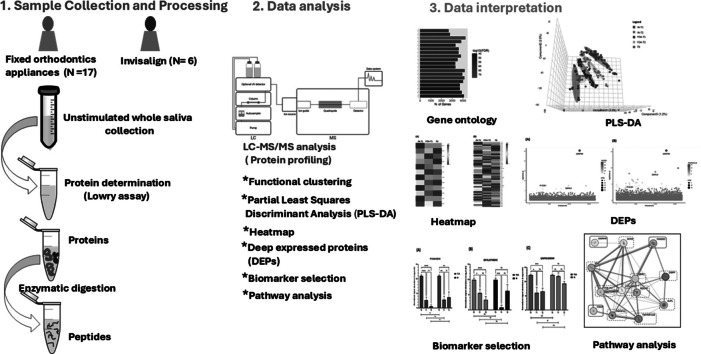

## Introduction

Orthodontic tooth movement (OTM) induces biological responses, altering periodontal ligament blood flow and triggering the release of mediators that initiate bone remodelling.^1^ While clinical outcomes of OTM are traditionally measured through aesthetic and functional parameters such as occlusion and tooth alignment, these changes often require extended periods to become visually apparent. Therefore, early detection methods are crucial for monitoring the effectiveness of OTM. Salivary proteins offer a non-invasive approach to monitor both the progression and potential adverse consequences of OTM. ^2^

Salivary biomarkers might be classified into 5 distinct categories: electrolytes, enzymes, hormones, immunoglobulins, and inflammatory mediators. A recent systematic review investigated the impact of orthodontic therapeutic interventions on these biomarker classifications. Regarding electrolyte alterations, orthodontic tooth movement was associated with decreased concentrations of calcium ions (Ca^2+^), phosphate ions (Pi^3^^-^), and potassium ions (K^+^), while simultaneously elevating sodium (Na^+^) and chloride (Cl^-^) levels. Enzymatic biomarkers, including alkaline phosphatase, lactate dehydrogenase, and matrix metalloproteinase (MMP)-8 and MMP-9, exhibited progressive increases throughout the treatment period. Hormonal analysis revealed diminished leptin concentrations alongside variable daily cortisol patterns during orthodontic intervention. The immunoglobulin profile, encompassing IgA, IgG, IgM, IgD, and IgE, demonstrated no statistically significant alterations, although salivary IgA exhibited inconsistent findings across different investigations. Inflammatory mediators, specifically soluble receptor activator of nuclear factor-kappa B ligand (sRANKL), osteoprotegerin (OPG), interleukin-1β (IL-1β), and prostaglandin-E2 (PGE-2), displayed temporal variations corresponding to different phases of orthodontic treatment, with pronounced changes following appliance activation. Nevertheless, given the substantially limited quality of available evidence, the systematic review concluded that orthodontic tooth movement exerted minimal measurable influence on salivary biomarker profiles.^3^

Salivary biomarkers, identified through advanced mass spectrometry techniques, provide diagnostic advantages due to their high sensitivity and specificity.^4^ Previous studies have documented changes in specific proteins involved in inflammation and bone remodeling in the saliva of orthodontic patients, including S100 calcium-binding protein A9 (S100-A9), immunoglobulin J chain, Ig alpha-1 chain C region, cysteine-rich secretory protein 3 precursor (CRISP-3),^5^ bone morphogenetic protein 4 (BMP4),^6^ and apolipoprotein E.^7^

Contemporary proteomic research has predominantly focused on comparative analyses between orthodontically treated populations and untreated control groups. However, limited scientific attention has been directed toward elucidating proteomic distinctions arising from different therapeutic approaches, specifically between conventional fixed orthodontic appliances (FOA) and clear aligner systems such as Invisalign (IN). A comprehensive systematic review revealed that FOA treatment induced more pronounced alterations in oral microbial communities relative to IN therapy. The investigation documented significant decreases in salivary pH values, total protein concentrations, and calcium levels among FOA patients, while simultaneously observing elevated glucose and amylase concentrations. Conversely, IN treatment demonstrated no measurable alterations in salivary flow rates or buffering capacity.^8^

Despite evidence indicating that both therapeutic modalities influence salivary biochemical profiles, substantial knowledge deficits persist regarding the specific biomarkers involved, the magnitude of compositional changes, and their implications for oral health maintenance. Furthermore, the potential utility of salivary markers as monitoring tools for treatment progression and therapeutic efficacy remains underexplored. Comprehensive research initiatives are critically needed to address these knowledge gaps and facilitate the development of personalized, evidence-based orthodontic tooth movement strategies.^9^ Therefore, the aim of this study was to identify potential salivary proteins that respond specifically to FOA and IN treatments.

## Methods

### Ethical approval

The Human Research Ethics Committee of Walailak University (Nakhon Si Thammarat, Thailand) approved the protocols for clinical treatments and saliva collection from orthodontic patients (protocol code WU-EC-DE-1-429-65, date of approval 30 March 2023). Written informed consent was obtained from each participant before any study-related procedures were performed. This study was conducted in compliance with the applicable ethical standards on human experimentation and with the Helsinki Declaration of 1975, as revised in 2013.^10^

### Study design and subjects

This prospective observational study aimed to compare salivary protein profiling in patients with FOA and IN. The participants were selected from a population of patients who were going to be treated for their malocclusion in the Center for Advanced Oral Health, International College of Dentistry, Walailak University, Bangkok, Thailand, between April 2023 and March 2024. Sample size estimation was performed using G Power software version 3.1.9.4 (Heinrich-Heine-Universität Düsseldorf, Düsseldorf, Germany). Conventional statistical guidelines classify standardised effect sizes according to established criteria: values between 0.2 and 0.5 represent small effects, 0.5 to 0.8 indicate moderate effects, while values exceeding 0.8 denote large effects.^11^ Within the context of human omics research, reported maximum standardised effect sizes typically fall within the range of 0.4 to 0.8.^12^ Based on the anticipated large standardised effect size (Cohen's d = 0.8), statistical power threshold of 80%, alpha significance criterion of 0.05, 2-group comparative design, longitudinal assessment across 3 temporal measurement intervals, and inter-measurement correlation coefficient of 0.50, statistical analyses established the minimum required sample size as 12 participants.^13^ The selection of participants was made on the bases of the following inclusion criteria: permanent dentition, adult age, and moderate to severe malocclusions.^14^ The following parameters were taken as exclusion criteria: history of previous orthodontic treatment, antibiotic therapy within the past 3 months, current systemic medication use, active smoking, diagnosis of systemic disease, and signs of gingivitis and/or periodontitis.

The orthodontic technique adopted for the treatment of each subject has been already chosen for each participant by the expert orthodontists, prior to the beginning of this research project. Seventeen subjects received fixed orthodontic appliances with metallic brackets of 0.022-inch slot size (American Orthodontics). The bonding process was performed using the same adhesive (BracePaste, American Orthodontics) for all patients, and treatment was initiated with 0.014-inch NiTi archwires (Highland Metals). Six subjects received clear aligners (Invisalign®, Align Technology Inc.). All participants received standardised oral hygiene instructions. Data collection and follow-up of the participants were performed in the following way. A proper informed consent form was signed by each participant during one of the preliminary appointments before beginning the actual treatment. Then, at the day scheduled for the beginning of the orthodontic treatment a salivary sample was taken from each participant preceding to the bonding procedure (T0). Other salivary samples were taken after periods of 3 (T1) and 6 months (T2). All the salivary samples were taken by the same operator.

### Collection and processing of saliva

Participants were instructed to rest for 15 minutes before saliva collection, which occurred between 9:00-12:00 am, and to refrain from consuming food and beverages for at least 1 hour prior to collection. A total of 5 mL of unstimulated whole saliva were collected by drooling into 50 mL sterile plastic centrifuge tubes. Immediately after collection, saliva samples were centrifuged at 10,000 × g for 15 minutes at 4°C to remove insoluble materials, cells, and debris. The supernatants were stored at -80°C for subsequent analyses. To minimise analytical variability and enhance the statistical robustness of omics dataset interpretation, triplicate technical measurements were conducted for each individual biological specimen.^15^

### Salivary sample processing for liquid chromatography-tandem mass spectrometry (LC-MS/MS) analysis

Protein concentration of saliva samples was determined using the Lowry assay with bovine serum albumin (Sigma-Aldrich) as a standard protein.^16^ Five micrograms of protein samples underwent in-solution digestion. Salivary proteins were dissolved in 10 mM ammonium bicarbonate (AmBic), and disulphide bonds were reduced using 5 mM dithiothreitol (DTT) in 10 mM AmBic at 60°C for 1 hour. Alkylation of sulfhydryl groups was performed using 15 mM iodoacetamide (IAA) in 10 mM AmBic at room temperature for 45 minutes in darkness. Proteins were then digested with sequencing-grade porcine trypsin (1:20 ratio) for 16 hours at 37°C. The resulting tryptic peptides were dried in a speed vacuum concentrator and resuspended in 0.1% formic acid for nano LC-MS/MS analyses.

### LC-MS/MS analysis and protein identification

Tryptic peptide samples were prepared for injection into an Ultimate3000 Nano/Capillary LC System (Thermo Scientific, UK) coupled to a ZenoTOF 7600 mass spectrometer (SCIEX). Briefly, one microliter of peptide digest was enriched on a μ-precolumn 300 μm i.d. × 5 mm C18 Pepmap 100, 5 μm, 100 Å (Thermo Scientific, UK), then separated on a 75 μm I.D. × 15 cm column packed with Acclaim PepMap RSLC C18, 2 μm, 100 Å, nanoViper (Thermo Fisher Scientific). The C18 column was housed in a thermostatted column oven maintained at 60°C. Solvents A and B, containing 0.1% formic acid in water and 0.1% formic acid in 80% acetonitrile, respectively, were supplied to the analytical column. A gradient of 5-55% solvent B was used to elute the peptides at a constant flow rate of 0.30 μL/min for 30 minutes. Mass spectrometry-derived raw datasets underwent computational processing through MaxQuant software (version 2.4.2.0) to facilitate protein identification and quantitative analysis. Spectral correlation against the Uniprot Homo sapiens reference database was accomplished using the integrated Andromeda search algorithm.^17^ Protein false discovery rate (FDR) was set at 1% and estimated using reversed search sequences. The maximum number of modifications per peptide was set to 5. The search utilized the *Homo sapiens* proteome FASTA file downloaded from Uniprot (March 31, 2024).

### Data visualisation and statistical analyses

The MaxQuant ProteinGroups.txt file was imported into Perseus version 1.6.6.0.^17^ Potential contaminants not corresponding to any UPS1 protein were removed from the dataset. ShinyGO 0.77 software was used for in-depth analysis of salivary proteins involved in biological processes, with graphical visualisation of enrichment.^18^ Visualisation and statistical analyses of the LC-MS data, including Partial Least Squares Discriminant Analysis (PLS-DA), heatmap generation, and analysis of variance (ANOVA) with post-hoc Tukey HSD (Honestly Significant Difference) test, were conducted using Metaboanalyst version 6.0, with a significance threshold of P-value ≤ .05.^19^ Additionally, as a complementary statistical approach, 2-way repeated measures ANOVA followed by Tukey's multiple comparisons test was performed using GraphPad Prism version 10.0 for Windows (GraphPad Software). A *P*-value ≤ .05 was considered statistically significant. The STRING (Search Tool for the Retrieval of Interacting Genes) database version 12.0 was used to investigate interactions with proteins related to OTM. ^20^ These proteins included alkaline phosphatase (ALPL), ^21^ osteocalcin (BGLAP) ^21^ collagen alpha-1 (COL1A1), collagen alpha-2 (COL1A2), ^22^ dentine sialophosphoprotein (DSPP),^23^ Sex-determining region Y-box 9 (SOX9), ^24^ osteopontin (OPN or SPP1), ^25^ tumor necrosis factor receptor superfamily member 11B or osteoprotegerin (TNFRSF11B or OPG), tumor necrosis factor receptor superfamily member 11 (TNFSF11 or RANK), ^26^ and fibronectin (FN1).^27^

## Results

### Characteristics of participants

A total of 23 patients (15 females and 8 males, aged 19-52 years; mean 28 ± 8.88 years) participated in this study. The FOA group (N = 17; 5 males, 12 females) had a mean age of 27 ± 8.86 years (range: 19-52 years). The IN group (N = 6; 3 males, 3 females) had a mean age of 32 ± 8.85 years (range: 19-46 years). All patients had a median crowding of 4 mm (range: 4-10 mm). Regarding malocclusion types,^14^ 70% of total patients presented with moderate crowding, while 30% exhibited severe crowding. In the FOA group, the median crowding was 4 mm (range: 4-10 mm), with 71% classified as moderate crowding and 29% as severe crowding. In the IN group, the median crowding was 4.5 mm (range: 4-8 mm), with 67% exhibiting moderate crowding and 33% severe crowding.

### Salivary protein profiles in response to FOA and IN at T0, T1, and T2

**Fig. 1:**
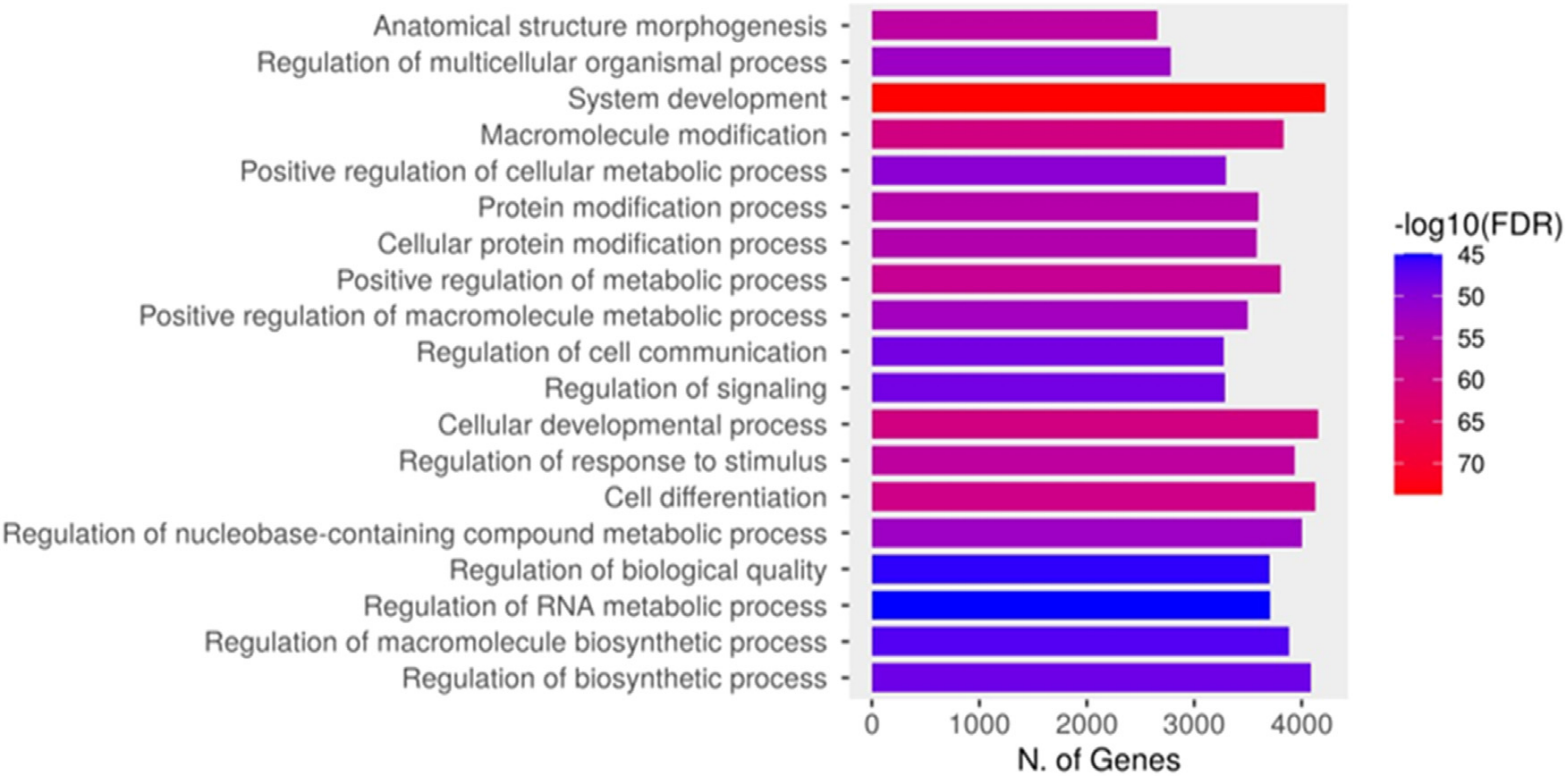
Functional classification of the identified proteins based on the biological process category of Gene Ontology (GO). The number of 17,603 DEPs involved in biological processes were identified in whole saliva of FOA and IN patients. Within each overarching biological process category, proteins were further classified into specific GO terms, presented on the y-axis, with the corresponding number of proteins indicated on the x-axis. Protein expression levels, represented as -log_10_(FDR), are indicated by a colours gradient ranging from blue (low expression) to red (high expression).

Comprehensive liquid chromatography-tandem mass spectrometry analysis of salivary protein constituents, integrated with gene ontology enrichment evaluation, provided mechanistic insights into biological processes activated during orthodontic tooth movement. This analytical strategy yielded the identification of 17,603 differentially expressed proteins (DEPs) associated with OTM. Functional annotation through ShinyGO biological process categorisation demonstrated that the characterized salivary proteins predominantly participated in system development, cellular developmental processes, cell differentiation, and biosynthetic regulation pathways (Figure 1).

**Fig. 2:**
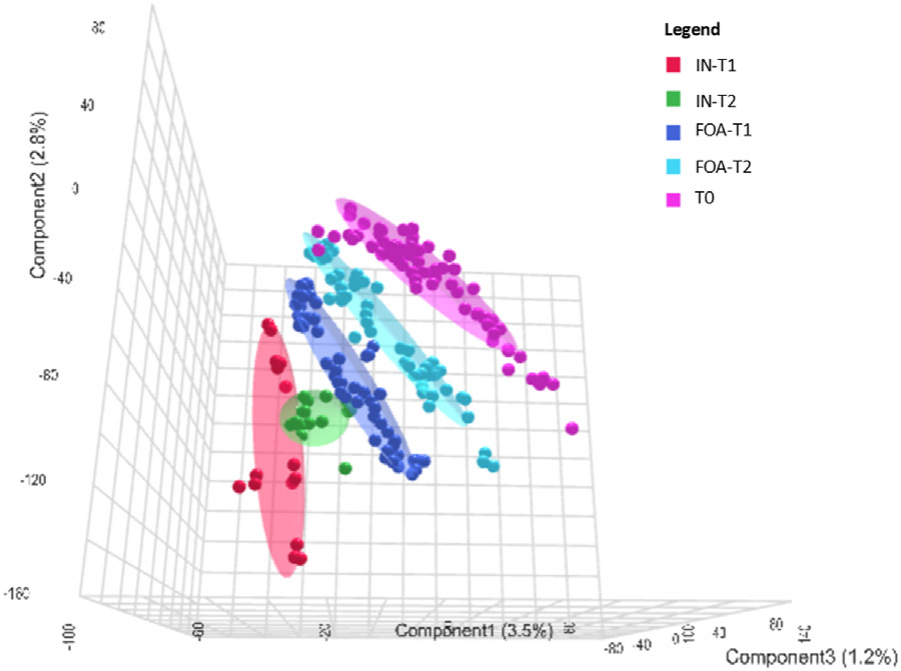
Three-dimensional partial least squares discriminant analysis (3D PLS-DA) of salivary proteins at T0, T1, and T2. The 3D PLS-DA plot captures variance between groups using the first 3 components. Component 1 explains 3.5% of the variance, component 2 explains 2.8%, and component 3 accounts for 1.2% of the variance. Different groups are represented by distinct colours with corresponding ellipsoids delineating the 95% confidence intervals of data distributions. The red, green, dark blue, light blue, and pink points represent individual saliva samples from 5 distinct observational conditions, emphasising the multivariate separation among them. Pink represents expressed proteins at T0 for both FOA and IN groups. Dark blue and light blue represent FOA at T1 and T2, respectively. Red represents IN at T1, while green denotes IN at T2. Notably, the 3D PLS-DA plots show comparable salivary protein profiles between the 2 treatment groups at baseline assessment (T0) whereas FOA and IN treatments significantly effect on salivary metabolites.

To elucidate the distinct effects of FOA and IN treatments on salivary proteomic signatures, PLS-DA was implemented. The analysis revealed comparable salivary protein profiles between treatment groups at baseline assessment (T0) (Figure 2), indicating equivalent metabolic status preceding orthodontic intervention initiation. However, distinct separation of protein profiles between the FOA and IN groups was observed at the T1 and T2 compared to T0. Within each treatment group, minimal separation of protein clusters was evident between T1 and T2. These findings indicate that FOA and IN treatments induce differential alterations in the salivary proteomic landscape over time.

### Identification of DEPs in FOA and IN at T0, T1, and T2

**Fig. 3:**
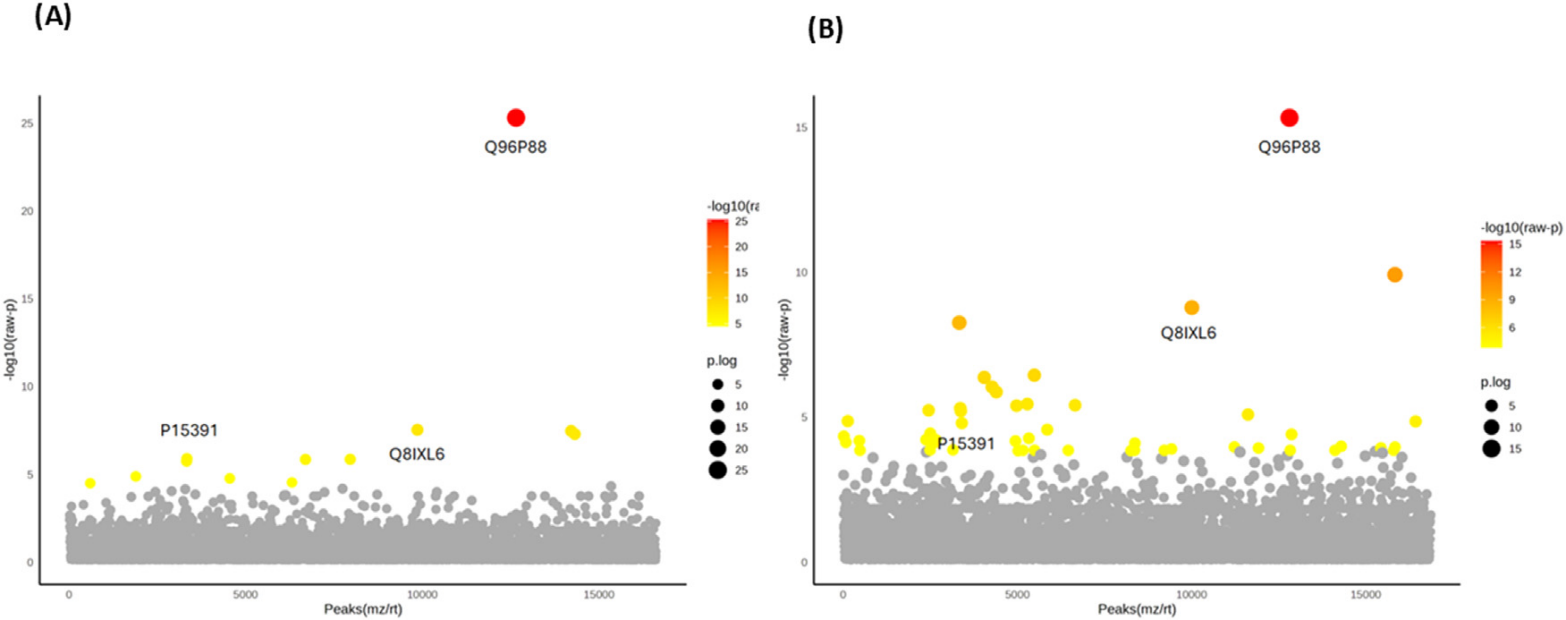
One-way ANOVA identification of salivary DEPs in response to orthodontic treatments. The plots depict one-way ANOVA results showing **(A)** the top 12 DEPs when comparing FOA and IN at T1 and **(B)** the top 50 DEPs when comparing FOA and IN at T2. Only 3 proteins—P15391 (CD19), Q8IXL6 (FAM20C), and Q96P88 (GNRHR)—were identified in both groups. Note: The Y-axis scale of the scatter plot was automatically adjusted by Metaboanalyst based on the range of -log_10_(raw P). Coloured dots (yellow to red spectrum) represent statistically significant proteins (*P* < .05), while gray dots indicate no significant difference in protein expression (*P* > .05).

**Fig. 4:**
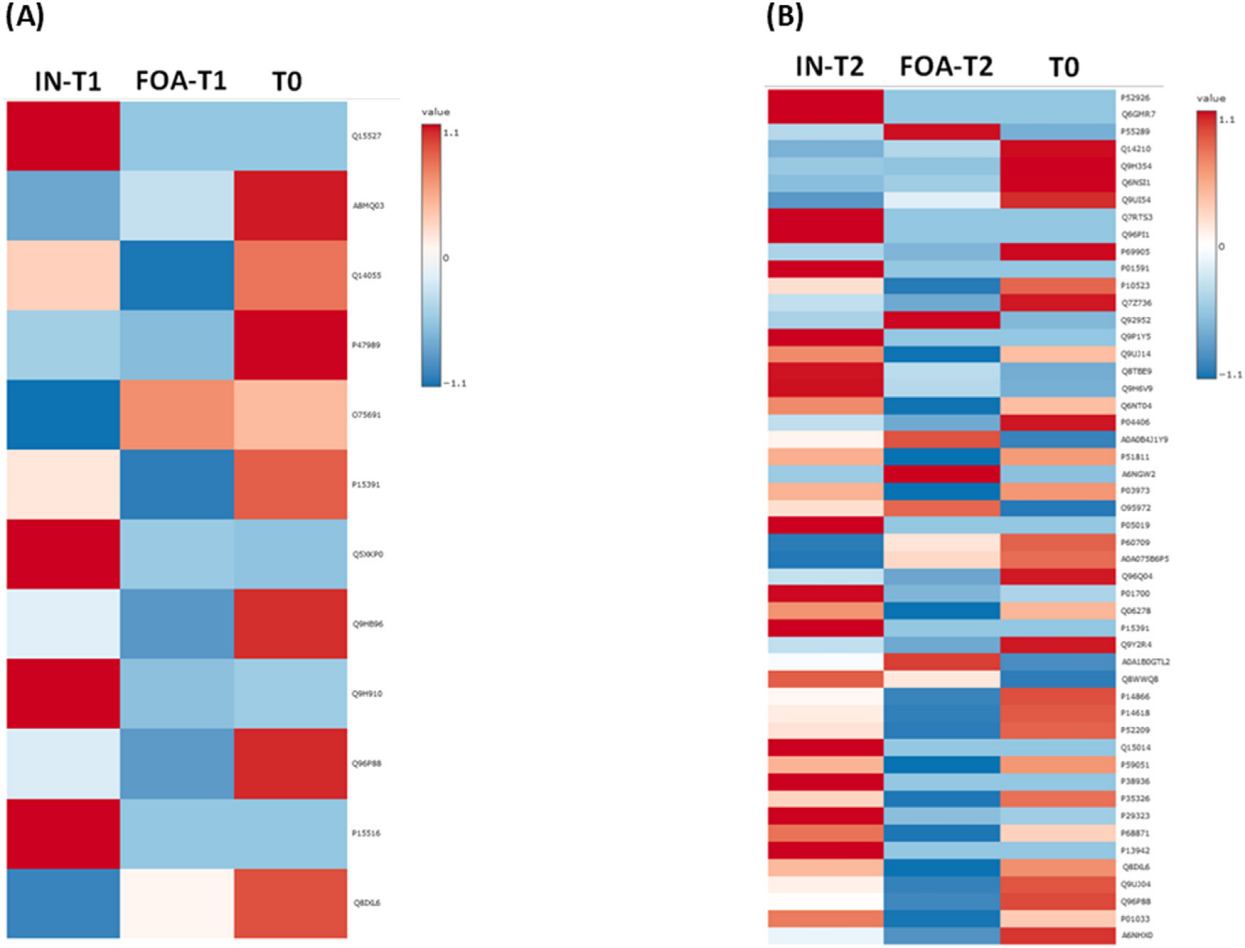
Heatmap analysis of DEPs in FOA and IN at T0, T1, and T2. **(A)** Heatmap of the top 12 proteins based on one-way ANOVA in FOA and IN at T1 compared to T0. **(B)** Heatmap of the top 50 proteins based on one-way ANOVA in FOA and IN at T2 compared to T0. The colour gradient from blue to red indicates the degree of expression from low to high.

Among the 17,603 DEPs, 12 were significantly differentially expressed between the FOA and IN groups at T1 (Figure 3A), while 50 DEPs were identified between the 2 groups at T2 (Figure 3B). Three proteins—P15391 [cluster of differentiation (CD)19)], Q8IXL6 [family with sequence similarity (FAM) 20C], and Q96P88 [Gonadotropin-releasing hormone receptor (GNRHR)]—were identified in both groups at both time points. Heatmap visualisation of expression trends across the 2 groups for the 12 and 50 DEPs based on ANOVA is shown in Figures 4A and 4B, respectively. The heatmap revealed that among the 12 DEPs responding to FOA and IN treatments at T1, 10 proteins exhibited decreased expression levels in the FOA group, while 3 proteins were downregulated in the IN group (Figure 4A). Figure 4B demonstrates that after 6 months of orthodontic treatment, 40 proteins were downregulated in the FOA group, while 14 proteins were downregulated in the IN group.

**Fig. 5:**
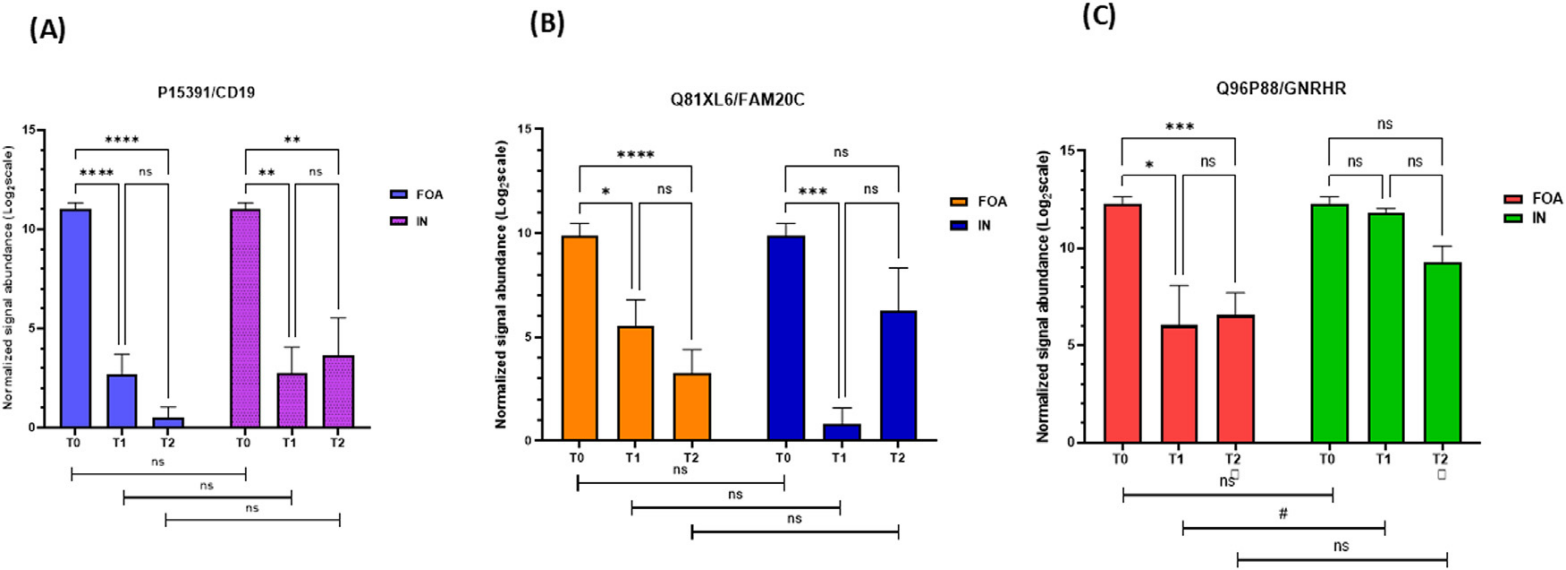
Bar graphs of CD19, FAM20C, and GNRHR expression in FOA and IN at T0, T1, and T2. These graphs represent the results of 2-way repeated measures ANOVA and Tukey's HSD test comparing the mean values of **(A)** P15391/CD19, **(B)** Q8IXL6/FAM20C, and **(C)** Q96P88/GNRHR from baseline over time between the FOA and IN groups. The y-axis represents the normalized peak intensity of proteins obtained from LC-MS/MS analysis. The x-axis represents 3 time points in each group. Each bar represents the mean (± SEM) of 3 different replicates.* *P* < .05, **P < .01, ***P < .001 and ****P < .0001 indicate levels of statistical significance between time points within groups, whereas ^#^*P* < .05 indicates FOA versus IN differences at the same time point.

We focused particularly on DEPs expressed in both FOA and IN groups. Two-way repeated measures ANOVA and Tukey's HSD tests demonstrated significant effects of time (*P* < .05) and group (*P* < .05) for CD19 (Figure 5A) and FAM20C (Figure 5B), but no significant time × group interaction (*P* > .05) for these proteins. Significant effects of time (P < .05), group (P < .05), and time × group interaction (P < .05) were observed for GNRHR (Figure 5C). Expression levels of CD19 and FAM20C were significantly lower (*P* < .05) after 3 months of FOA or IN treatment compared to baseline. However, no further significant differences (*P* > .05) in the expression of these proteins were observed at the 6-month follow-up. Significant reduction (*P* < .05) in GNRHR expression was observed after 3 months in the FOA group, but no significant differences (*P* > .05) were found across time points in the IN group. GNRHR expression did not significantly change at T2 compared to T1 in the FOA group. Notably, a significant difference (*P* < .05) in GNRHR expression was observed between the FOA and IN groups at T1.

### Pathway analysis with the STRING database

**Fig. 6:**
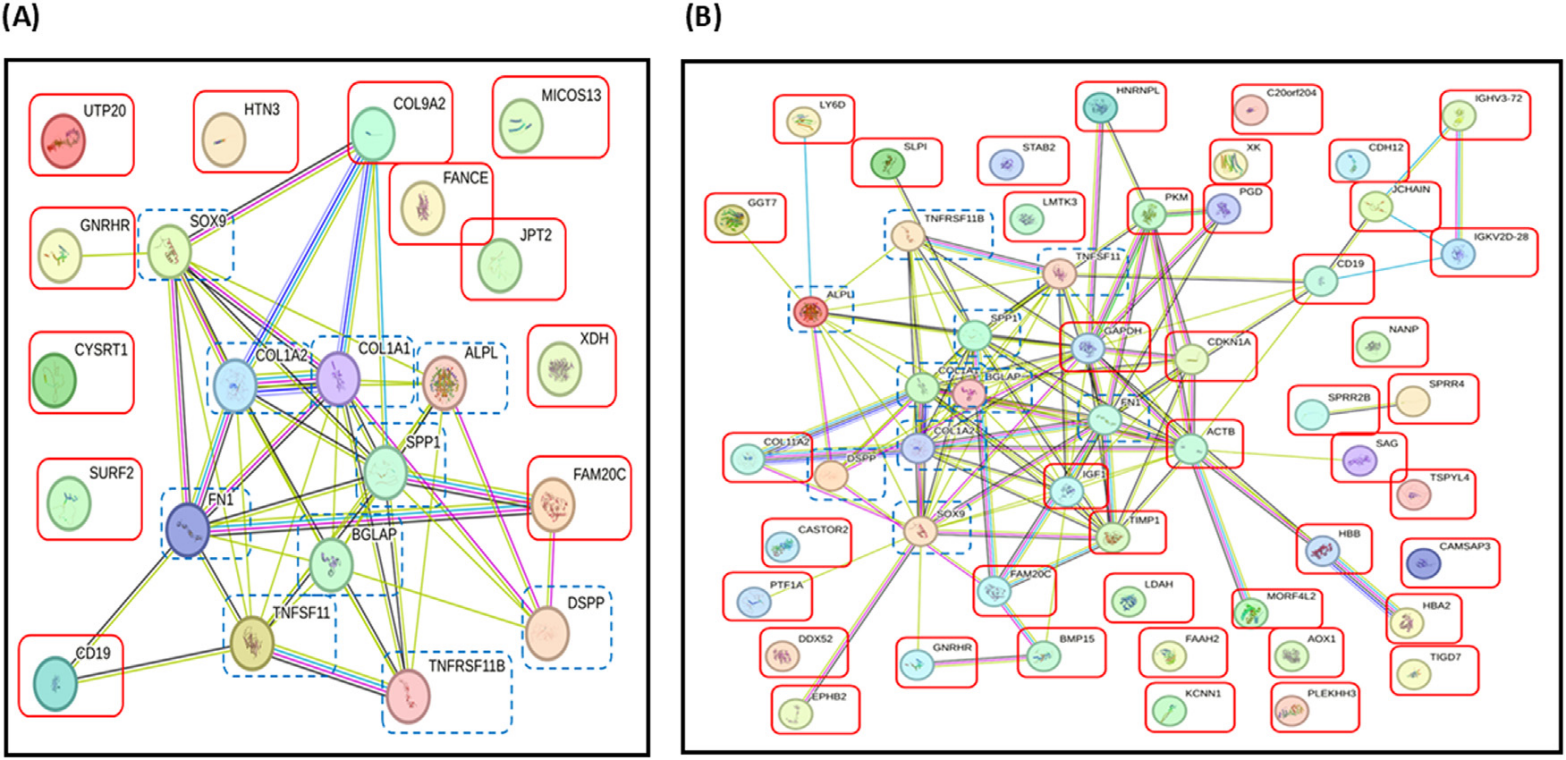
Interaction networks of top 12 DEPs **(A)** in FOA and IN at T1 and top 50 DEPs **(B)** in FOA and IN at T2. The STRING database demonstrated associations between certain proteins among these DEPs (red boxes) and proteins related to OTM (dashed blue boxes), including ALPL (alkaline phosphatase), BGLAP (osteocalcin), COL1A1 (collagen alpha-1), COL1A2 (collagen alpha-2), DSPP (dentine sialophosphoprotein), TNFSF11 (tumour necrosis factor receptor superfamily member 11 or RANK), TNFRSF11B (tumour necrosis factor receptor superfamily member 11B or osteoprotegerin,OPG), SOX9 (sex-determining region Y-box 9), SPP1 (osteopontin, OPN), and FN1 (fibronectin). Proteins are represented by coloured nodes, and interactions are represented by edges. Lines between nodes (edges) represent the type of evidence used in predicting the associations: co-occurrence evidence (blue), neighbourhood evidence (green), experimental evidence (purple), database evidence (light blue), and co-expression evidence (black).

**Fig. 7:**
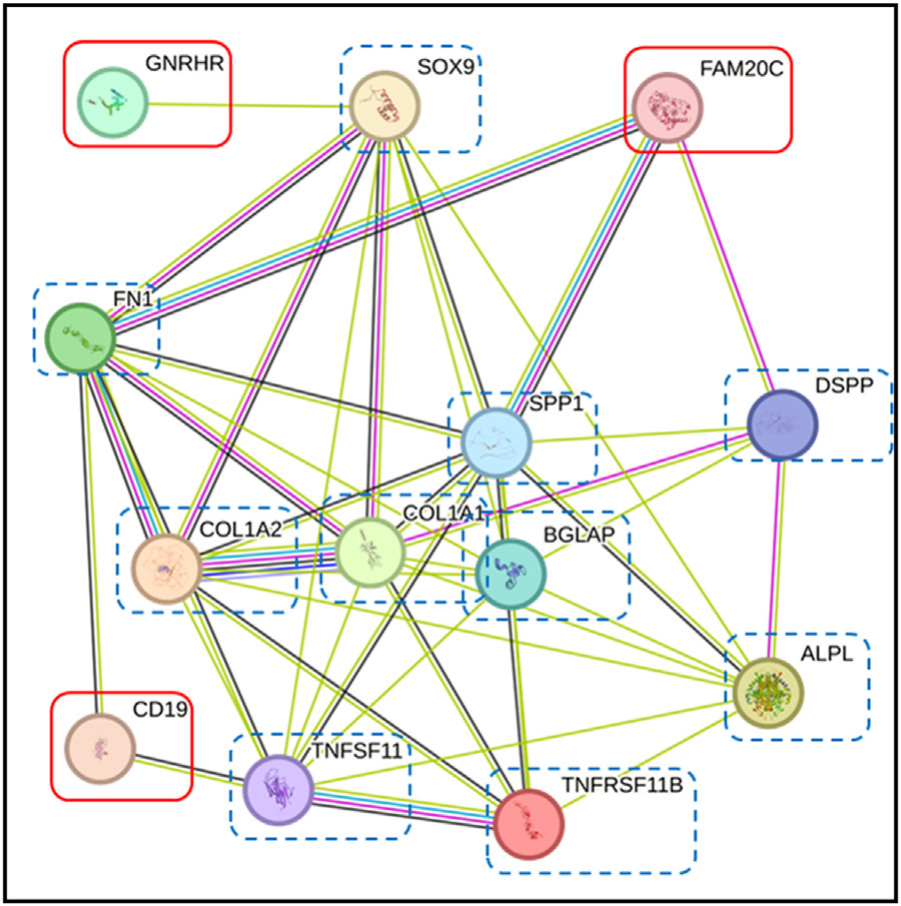
Interaction networks of CD19, FAM20C, and GNRHR in FOA and IN at T1 and T2. The STRING database demonstrated associations between CD19, FAM20C, and GNRHR (red boxes) and proteins related to OTM (dashed blue boxes). CD19 was directly linked to FN1 and TNFSF11. FAM20C directly associated with FN1, SPP1, and DSPP. GNRHR indirectly interacted with FN1, ALPL, COL1A1, COL1A2, SPP1, and TNFRSF11 via SOX9. Proteins are represented by coloured nodes, and interactions are represented by edges. Lines between nodes (edges) represent the type of evidence used in predicting the associations: co-occurrence evidence (blue), neighbourhood evidence (green), experimental evidence (purple), database evidence (light blue), and co-expression evidence (black).

To elucidate the potential roles of DEPs found in FOA and IN groups, we assessed their interactions with proteins involved in OTM using the STRING database. The 12 DEPs (Figure 6A) and 50 DEPs (Figure 6B) interacted with alkaline phosphatase (ALPL), osteocalcin (BGLAP), collagen alpha-1 (COL1A1), collagen alpha-2 (COLA2), dentine sialophosphoprotein (DSPP), sex-determining region Y-box 9 (SOX9), tumour necrosis factor receptor superfamily member 11B (TNFRSF11B) or osteoprotegerin (OPG), osteopontin (SPP1 or OPN), tumour necrosis factor receptor superfamily member 11 (TNFSF11) or RANK and fibronectin (FN1). Notably, the 3 top proteins expressed in both groups—CD19, FAM20C, and GNRHR—demonstrated relationships with proteins involved in OTM. CD19 was directly linked to fibronectin and RANK. FAM20C directly associated with fibronectin, OPN, and DSPP, while GNRHR indirectly interacted with alkaline phosphatase, COL1A1, COLA2, fibronectin, OPN, and RANK via SOX9 (Figure 7).

## Discussion

In this study, top DEPs were selected based on the P-values from ANOVA and post hoc analyses. STRING database analysis revealed associations between these DEPs and proteins related to OTM, including alkaline phosphatase, osteocalcin, collagen alpha-1, collagen alpha-2, dentine sialophosphoprotein, SOX-9, OPN, OPG, RANK, and FN1. Among these DEPs, CD19, FAM20C, and GNRHR were selected for further investigation based on their significant expression changes in both treatment groups at T1 and T2.

Orthodontic tooth movement represents a form of sterile inflammatory response, wherein T and B cells, contribute significantly to alveolar bone resorption processes. B cells, in conjunction with T lymphocytes and additional leukocyte subsets, synthesize receptor activator of nuclear factor kappa B (NF-κβ) ligand (RANKL), a critical signalling molecule that promotes osteoclastic activation and subsequent bone resorption at compression sites during orthodontic therapy.^1^ Mechanical forces applied during orthodontic treatment are detected by osteoblastic lineage cells, which respond by secreting various cytokines. Among these inflammatory mediators, RANKL emerges as the predominant factor facilitating osteoclast differentiation and maturation. The RANKL-RANK signalling cascade represents a fundamental mechanism underlying orthodontic-induced bone remodelling. Ligand-receptor interaction between RANKL and RANK triggers osteoclastogenesis via downstream transcription factor activation, while OPG functions as a competitive inhibitor within this regulatory network, thereby modulating bone remodelling dynamics through the integrated RANKL/RANK/OPG system.^26^

Contemporary research has identified B-lymphocyte-associated proteins, particularly B-cell lymphoma 6 (BCL6)—a transcriptional suppressor governing B-lymphocyte maturation—as regulatory factors in osteoclast differentiation and periodontal ligament cellular proliferation under mechanical loading conditions. BCL6 prevents excessive osteoclast formation and hyperactivity within compression zones, consequently minimising alveolar bone destruction and potentially preserving periodontal structural integrity during orthodontic intervention.^28^ The pronounced suppression of B-lymphocyte developmental pathways, as evidenced through CD19 downregulation within our experimental samples at both T1 and T2 timepoints and confirmed through STRING network analysis, represented an unanticipated finding. The CD19 surface antigen constitutes a 95-kilodalton transmembrane glycoprotein within the immunoglobulin superfamily, serving critical functions in B-lymphocyte signalling threshold establishment through regulation of both receptor-dependent and receptor-independent cellular communication pathways.^29^ Nevertheless, definitive evidence supporting direct CD19 participation in orthodontically-induced bone remodelling remains sparse. A previous murine investigation documented peak CD19 expression at day 3 following orthodontic activation, with subsequent expression decline.^30^ These collective findings emphasising immune system involvement in orthodontic mechanisms have prompted the introduction of a novel research domain termed immunoorthodontics.^31^

STRING analysis in our study suggests associations between CD19, FN1, and RANK. Currently, little is known about the relationships among these proteins in OTM. Extracellular matrix components, particularly FN1, gradually increase in the pressured side and decrease in the tension side during orthodontic therapy. ^32^ Fibronectin/β1-integrin inhibits osteoclast formation by preventing pre-osteoclast fusion. Therefore, increased FN1 expression during orthodontic treatment may contribute to controlled bone remodelling and tooth movement.^33^ Collectively, our results suggest an important contribution of CD19 in modulating OTM. Although the precise interplay between CD19 and orthodontic forces requires further elucidation, our findings demonstrate a tangible link between the 2. A decrease in CD19 might be associated with reduced osteoclastogenesis, potentially downregulating excessive bone resorption and contributing to the maintenance of a healthy alveolar bone during OTM.^28^

FAM20C is a member of the family with sequence similarity 20, consisting of 3 members: FAM20A, FAM20B, and FAM20C. FAM20C is highly expressed in chondrocytes, osteoblasts, osteocytes, odontoblasts, ameloblasts, and cementoblasts, as well as in dentine, enamel, and bone matrices. FAM20C plays an important role in the formation of these mineralised tissues and subsequent mineralisation processes. ^34^ FAM20C participates in mineralisation mechanisms via post-translational modification of secretory calcium-binding phosphoproteins (non-collagenous matrix components) predominantly distributed within osseous and dental tissues. This protein family encompasses small integrin-binding ligand N-linked glycoproteins (SIBLINGs), including dentin matrix protein 1 (DMP1), bone sialoprotein (BSP), OPN, matrix extracellular phosphoglycoprotein (MEPE), and DSPP. These SIBLING molecules exhibit substantial calcium-binding capacity and orchestrate calcium phosphate deposition as hydroxyapatite within skeletal and dental structures.^35^

FAM20C demonstrates significant involvement in osseous metabolic processes, modulating osteoblastic proliferation and contributing to osteoclastic bone resorption activities. Genetic alterations in FAM20C compromise mineralisation capacity and disrupt bone mineral homeostasis, as exemplified in Raine syndrome—a pathological condition characterized by osteosclerotic changes and associated developmental abnormalities. These clinical manifestations underscore FAM20C's essential function in skeletal metabolic regulation.^36^ Nevertheless, understanding of FAM20C's specific role in OTM remains incomplete. Network analysis conducted in this investigation revealed functional associations between FAM20C and OPN. OPN represents a pleiotropic protein recognized as fundamental to bone remodelling dynamics, tissue mineralisation, and periodontal tissue adaptation during orthodontic intervention.^25^ This protein demonstrates widespread expression throughout periodontal remodelling processes, establishing its potential utility as a tissue response biomarker for orthodontic treatment monitoring.^37^ Salivary BMP4 detection following fixed orthodontic appliance installation indicates its significant contribution to alveolar bone formation and tissue homeostatic maintenance during tooth movement.^6^ Previous research has identified both OPN and BMP4 as functional substrates for FAM20C enzymatic activity.^38^ OTM exhibits mechanistic similarities to developmental tooth formation processes, both requiring coordinated bone turnover.^39^ Consequently, our findings suggest that orthodontic force application may trigger cascading events analogous to developmental bone remodelling mechanisms. FAM20C potentially phosphorylates both OPN and BMP4, subsequently initiating diverse biological processes including bone resorption during orthodontic treatment.

GNRHR, localised within pituitary tissue, binds gonadotropin-releasing hormone (GnRH), subsequently stimulating luteinising hormone and follicle-stimulating hormone secretion. These pituitary hormones target gonadal tissues to promote sex hormone synthesis, including testosterone, oestrogen, and progesterone.^40^ GNRHR may influence orthodontic tooth movement through oestrogen-mediated effects on bone remodelling regulation. Elevated oestrogen concentrations inhibit bone resorption while promoting osteogenesis, potentially decelerating tooth movement velocity. Conversely, reduced oestrogen levels, such as those occurring during lactation, may accelerate orthodontic tooth movement rates.^41^

Our study suggests indirect interactions between GNRHR and certain OTM-related proteins, including alkaline phosphatase, collagen alpha-1, collagen alpha-2, fibronectin, OPN, and RANK via SOX9. To date, SOX9 has primarily been studied in tooth development, with few investigations reporting its possible role in OTM.^42^ A meta-analysis suggests that SOX9, parathyroid hormone-related protein, and Indian Hedgehog protein play vital roles in directly regulating type II collagen genes and transducing applied forces to bone and teeth during OTM.^43^ According to recent research, application of orthodontic forces results in timely and localised release of several cytokines that work in concert to initiate and sustain bone and periodontal tissue remodelling processes. Elevated concentrations of pro-inflammatory cytokines, including interleukin-1β, tumour necrosis factor-alpha (TNF-α), and interleukin-6, have been associated with osteoclast stimulation.^44^ A previous study showed that SOX9, as a downstream target of TNF-α, plays an important role in dental pulp inflammation and immune responses.^45^ However, conclusive evidence regarding the relationship between orthodontic force and pulp tissue in humans remains elusive. Nevertheless, tooth movement involves cell damage, inflammation, and wound healing processes, which may affect dental pulp health.^46^

Alterations in alkaline phosphatase activity serve as a biological marker of cellular responses in periodontal tissues during OTM. Multiple studies have demonstrated significant elevation in alkaline phosphatase levels from baseline following the application of orthodontic forces.^47^^,^^48^ Concurrently, RANKL concentrations in gingival crevicular fluid exhibit marked increases during active tooth movement.^49^ These findings indicate that PDL cells subjected to mechanical stress promote osteoclastogenesis through enhanced RANKL expression, facilitating the bone resorption phase of OTM.^26^ OPN and RANKL collectively work to induce the bone resorption in response to compressive forces. In this process, OPN functions synergistically with RANKL to mediate bone resorption in response to compressive forces applied during OTM. Furthermore, tension forces during OTM trigger the upregulation of several osteogenic markers including OPN, alkaline phosphatase, collagen type I, osteocalcin, and BSP through signalling cascades mediated by extracellular signal-regulated kinase (ERK) and p38 mitogen-activated protein kinase (MAPK) pathways.^25^

Zhang and colleagues^7^ previously characterised apolipoprotein E (ApoE) as a promising salivary biomarker for OTM monitoring. Our current findings present contrasting results, as ApoE was not detected among DEPs during orthodontic therapeutic interventions. These discordant outcomes may reflect methodological variations in experimental design and salivary sample processing protocols employed in our investigation. Nevertheless, the likelihood of undetected technical errors resulting in false-negative findings within our proteomic analyses appears minimal, given the implementation of liquid chromatography-tandem mass spectrometry—a highly sensitive analytical technique for protein detection.^4^

Existing literature has predominantly examined fixed appliance and clear aligner effects on oral microbiota and salivary parameters.^8^ Limited knowledge exists regarding alternative orthodontic treatment impacts on salivary biomarkers. Removable orthodontic treatment demonstrated non-significant salivary IgA changes at 3 months compared to baseline, with significant increases observed at 6 months. Notably, salivary IgA levels remained comparable between fixed and removable orthodontic groups at both 3 and 6-month intervals.^50^ Removable orthodontic intervention increased salivary lactate dehydrogenase and alkaline phosphatase levels after 1 month, with continued elevation at 3 months.^51^

### Clinical Implications

Prospective clinical trials incorporating larger sample sizes represent the optimal approach for biomarker verification and validation phases. The proposed utility of CD19, FAM20C, and GNRHR as salivary biomarkers requires validation through expanded patient numbers. To our knowledge, this investigation represents the inaugural study proposing these proteins as orthodontic treatment monitoring biomarkers. While substantial knowledge gaps persist regarding CD19, FAM20C, and GNRHR roles in orthodontic tooth movement, this study contributes valuable insights to current understanding. Clinical implementation of CD19, FAM20C, and GNRHR expression monitoring during orthodontic interventions may provide valuable biomarker resources for predicting treatment progression and therapeutic outcomes.

### Limitations

Several methodological constraints characterise this investigation. The primary limitation involves unequal sample distribution, with reduced participant numbers in the Invisalign treatment group. Biomarker development conventionally progresses through 3 sequential phases: discovery, verification, and validation. This investigation employed a cross-sectional design with limited sample sizes appropriate for the initial discovery phase of biomarker development. The discovery phase objectives encompass comprehensive identification of potential biomarker candidates through extensive, untargeted proteomic analysis aimed at detecting and quantifying maximal protein diversity. Subsequently identified candidate biomarkers undergo evaluation in verification and validation phases. Economic constraints typically necessitate restricted sample sizes during this initial phase, which involves putative peptide identification through computational matching of experimental tandem mass spectrometry spectra against predicted spectral databases.^52^ However, published guidelines suggest that 6 participants per group may provide adequate statistical power for robust biomarker discovery investigations.^53^ Furthermore, minimum reporting standards for analytical chemistry in metabolomic research recommend a minimum of 3 biological replicates—as implemented in our study—with 5 replicates representing the preferred standard.^54^

A second limitation encompasses demographic imbalances, specifically gender distribution within the FOA group and age disparities between FOA and IN groups. Several analytical strategies can mitigate sex-related confounding effects in salivary protein analysis using LC-MS/MS with constrained sample sizes, including multivariate approaches such as principal component analysis (PCA) or PLS-DA to visualise and evaluate overall protein expression patterns. These techniques facilitate identification of key differentiating proteins and assessment of sex-related data influences.^52^^,^^55^ Collectively, the current evidence foundation remains limited, necessitating well-designed randomised controlled trials with expanded sample sizes to validate our findings.

The third methodological constraint involves the potentially insufficient 6-month observation period for comprehensive salivary biomarker monitoring, which may restrict detection of long-term adaptive changes. While certain salivary biomarkers demonstrate rapid responsiveness to orthodontic forces, others may require extended periods to manifest significant alterations. Orthodontic tooth movement encompasses 3 distinct phases: the initial phase (characterised by immediate, rapid movement occurring 24-48 hours post-force application), the lag phase (lasting 20-30 days with minimal tooth displacement), and the post-lag phase (involving accelerated movement coinciding with bone remodelling, typically commencing around day 40).^56^ Consequently, numerous investigations focus on early orthodontic treatment stages when tooth movement activity peaks.^3^

Limited research has evaluated long-term orthodontic effects on salivary biomarkers. Matrix-assisted laser desorption/ionisation time-of-flight mass spectrometry (MALDI-TOF-MS) and Western blot analyses demonstrated significant salivary ApoE level changes following 2 and 12 months of orthodontic treatment.^7^ The inflammatory marker, myeloid-related protein (MRP)-8/14, exhibited significantly elevated levels after 3, 6, and 9 months of treatment compared to baseline values; however, 12-month levels showed no statistical difference from baseline.^57^ These studies collectively highlight ongoing controversy regarding long-term orthodontic effects on salivary biomarkers. Future research requires longitudinal investigations extending beyond 6 months to provide comprehensive understanding of salivary protein dynamics throughout complete orthodontic treatment courses.

## Conclusion

In summary, our findings demonstrate that different orthodontic treatment modalities—fixed orthodontic appliances and Invisalign systems—influence salivary proteomic profiles after 3-6 months of treatment. Comparison of protein expression in saliva between the 2 treatment approaches revealed 12 and 50 differentially expressed proteins after 3 and 6 months of treatment, respectively. Downregulation of CD19, FAM20C, and GNRHR was observed in both treatment groups. The 3 salivary proteins associated with proteins related to orthodontic tooth movement.

## Conflicts of interest

None disclosed.

## Author contributions

Conceptualisation, S.K., R.C., and S.R.; software, S.R. and S.K.; data collection, R.C. and S.K.; data analysis, S.K. and S.R.; writing—original draft preparation, R.C., S.R., and S.K.; writing—review and editing, R.C., S.R., and S.K.; funding acquisition, S.K. All authors have read and agreed to the published version of the manuscript.

## Ethics statement and consent to participate

The clinical treatments and saliva collection from orthodontic patients were conducted in accordance with the Declaration of Helsinki and approved by the Ethics Committee of Walailak University (protocol number WU-EC-DE-1-429-65, date of approval 30 March 2023).

## Funding

This study was supported by the International College of Dentistry, Walailak University, Bangkok, Thailand (grant number WUICD 2564).

## Data availability

The data that support the findings of this study are deposited with the ProteomeXchange Consortium (http://proteomecentral.proteomexchange.org) via the jPOST partner repository (https://jpostdb.org) with the data set identifier JPST003758 and PXD062882 (preview URL for reviewers: https://repository.jpostdb.org/preview/162480563467fbcaa19ba96, Access key: 6601).
